# Developing a measure of provider adherence to improve the implementation of behavioral health services in primary care: a Delphi study

**DOI:** 10.1186/1748-5908-8-19

**Published:** 2013-02-13

**Authors:** Gregory P Beehler, Jennifer S Funderburk, Kyle Possemato, Christina L Vair

**Affiliations:** 1 VA Center for Integrated Healthcare, VA WNY Healthcare System, Buffalo, NY, USA; 2 School of Nursing, University at Buffalo, The State University of New York, Buffalo, NY, USA; 3 School of Public Health and Health Professions, University at Buffalo, The State University of New York, Buffalo, NY, USA; 4 VA Center for Integrated Healthcare, Syracuse VA Medical Center, Syracuse, NY, USA; 5 Department of Psychology, Syracuse University, Syracuse, NY, USA; 6 Department of Psychiatry, University of Rochester, Rochester, NY, USA; 7 Department of Psychology, University of Colorado, Colorado Springs, CO, USA

**Keywords:** Primary healthcare, Mental health services, Guideline adherence, Delphi technique

## Abstract

**Background:**

The integration of behavioral health services into primary care is increasingly popular, yet fidelity of implementation in this area has been infrequently assessed due to the few measurement tools available. A sentinel indicator of fidelity of implementation is provider adherence, or utilization of prescribed procedures and engagement in model-specific behaviors. This study aimed to develop the first self-report measure of behavioral health provider adherence for co-located, collaborative care, a commonly adopted model of behavioral health service delivery in primary care.

**Methods:**

A preliminary 56-item measure was developed by the research team to represent critical components of adherence among behavioral health providers. To ensure the content validity of the measure, a modified Delphi study was conducted using a panel of co-located, collaborative care model experts. During three rounds of emailed surveys, panel members provided qualitative feedback regarding item content while rating each item’s relevance for behavioral health provider practice. Items with consensus ratings of 80% or greater were included in the final adherence measure.

**Results:**

The panel consisted of 25 experts representing the Department of Veterans Affairs, the Department of Defense, and academic and community health centers (total study response rate of 76%). During the Delphi process, two new items were added to the measure, four items were eliminated, and a high level of consensus was achieved on the remaining 54 items. Experts identified 38 items essential for model adherence, six items compatible (although not essential) for model adherence, and 10 items that represented prohibited behaviors. Item content addressed several domains, but primarily focused on behaviors related to employing a time-limited, brief treatment model, the scope of patient concerns addressed, and interventions used by providers.

**Conclusions:**

This study yielded the first content valid self-report measure of critical components of collaborative care adherence for use by behavioral health providers in primary care. Although additional psychometric evaluation is necessary, this measure may assist implementation researchers in clarifying how provider behaviors contribute to clinical outcomes. This measure may also assist clinical stakeholders in monitoring implementation and identifying ways to support frontline providers in delivering high quality services.

## Background

Despite advances in the treatment of behavioral health conditions in primary care, there remains a gap between treatment outcomes demonstrated in randomized clinical trials and the outcomes observed in real-world primary care clinics. Recognition of this gap has spurred numerous initiatives to improve the integration of empirically supported behavioral health services into the primary care setting. These healthcare integration strategies have been described in a number of related models [1]. For example, within the United States (US) Department of Veterans Affairs (VA) healthcare system, Primary Care-Mental Health Integration (PC-MHI) refers to the overarching goal of improving the health of primary care patients by offering mental and behavioral health services in primary care. One particular form of PC-MHI is co-located, collaborative care (CCC) in which behavioral health providers (BHPs) are embedded within primary care clinics to support the primary care team in the identification and treatment of common behavioral and mental conditions [2]. BHPs provide focused functional assessments, targeted interventions, and referral to specialty mental health services using a brief treatment model [3].

CCC has been employed regionally in VA since as early the 1990s with the mandate to implement CCC nationally since 2008 [4]. This model is popular outside of the VA as well, with 70% of community primary care clinics funded by the Health Resources and Services Administration using CCC [5]. Yet because CCC draws from several foundational models originally described by Strosahl [6], Cummings *et al.*[7], and Blount [8], there is variation in how the main components of CCC are implemented both across and within healthcare systems [9,10]. This model variability and inconsistent use of terminology used to describe CCC models [11] creates a challenge for healthcare administrators, frontline providers, and researchers. For example, in spite of the rapid growth in popularity of CCC models, the evidence base for the clinical effectiveness of CCC models has lagged considerably. Several studies conducted in the US Department of Defense (DOD) family medicine clinics found that patients who received four sessions or less of brief behavioral treatment from a BHP showed clinically significant decreases in distress symptoms and improved overall functioning [12,13]. Additionally, a recent longitudinal study suggested that the beneficial clinical effect was sustained at two years post-treatment [14]. Although these studies are limited by a lack of controlled comparisons, they provide early support for CCC and a rationale for conducting more rigorous studies through randomized trials.

There additionally remains a dearth of empirical studies describing how CCC models are typically implemented in primary care. This fact is striking given that CCC is a complex intervention that can be challenging for BHPs to implement due to a variety of influential patient, provider, clinic, and system level variables [15]. The functional roles and competencies of BHPs are quite broad [16] and although comprehensive educational and training programs have been described [17], they are relatively limited in number. With growing emphasis on understanding how complex healthcare interventions are implemented and disseminated, fidelity assessment is becoming an essential component of research in mental health services delivery [18]. Fidelity refers to the degree to which an intervention is implemented as intended [19], and is reflected in a variety of implementation frameworks and evaluation models [20,21]. Fidelity assessment is valuable on several levels, in that it improves confidence in conclusions drawn about the impact of an intervention and ensures a standardized dose of treatment [22]. Fidelity assessment can also provide an opportunity to understand which aspects of a program can be linked to clinical outcomes [18].

However, as reflected by a report from the Agency for Healthcare Research and Quality on the evidence to support integration of behavioral health and primary care [23], research into treatment fidelity for CCC models tends to be de-prioritized. To date, very few studies have attempted to formally assess fidelity in CCC and each has used a different approach to data capture. Shiner *et al.*[24] used a modified quality improvement checklist to assess which components of CCC best predicted improved patient outcomes. Their measure was completed by clinic coordinators and aimed to describe the function of the clinic as a whole, including clinic staffing characteristics, scheduling procedures, as well as several BHP clinical behaviors. Results from this study indicated six aspects of their CCC model predicted improvements in depression treatment outcomes. These aspects reflected the model components related to easily accessible mental healthcare based on brief, time-limited treatments. Although this study was valuable in providing empirical support for some CCC model components, the instrument used for assessing fidelity was specific to their regionally implemented VA model of CCC, rather than the variety of CCC models available, and did not consider the wide range of particular provider behaviors that could impact fidelity.

A subsequent pilot study also conducted in the VA was designed to determine the feasibility of using patient exit interviews to assess fidelity to another regionally-implemented model of CCC [25]. Patients were interviewed immediately following their CCC appointments, thereby acting as informants of BHP clinical behaviors. Results from this study suggested that providers completed about 64% of model-prescribed behaviors. Patient exit interview questions were easily understood by participants, minimally burdened BHPs, and introduced only minor changes in patient flow with CCC clinics. However, a key limitation to the exit interview approach was the need for interviewers to be readily available in primary care clinics. Additionally, although fidelity estimates generated from this approach were encouraging, these preliminary estimates need to be interpreted with caution due to the limited sample size. Finally, three additional VA studies reported on programmatic evaluations of CCC clinics by analyzing fidelity related metrics available in electronic medical records and administrative data. These metrics included CCC encounter length, frequency of encounters, mental health conditions diagnosed by BHPs, and clinical interventions used by BHPs [10,26,27]. Results from these studies were encouraging, and generally confirmed that BHPs were providing brief, time-limited treatment to patients presenting with a broad range of diagnoses. BHPs tended to provide patient education, supportive therapy, or behavioral activation as the most frequent interventions [10]. However, these chart review studies were unable to provide more detailed information regarding typical BHP behaviors both in relation to patients (such as assessment procedures used or approaches to referral management) and with primary care teams (approaches to care coordination with primary care providers).

Although proxy measures of clinical behaviors are imperfect, they may provide a more feasible approach to assessing fidelity than direct observation [28]. The proxies used in the above studies (clinic coordinator reports, patient reports, and chart review) all fail to gather self-reported data from the BHPs themselves who may be best able to describe the wide range of typical behaviors and patterns not easily measured using other approaches. To address the limitations of previous work regarding fidelity assessment for CCC, the aim of this study was to develop the first self-report measure of BHP fidelity to the CCC model by conducting a modified Delphi study. Because CCC has been conceptualized differently across a number of settings, our goal in employing a Delphi study with a diverse panel of content experts was to develop agreement on fundamental BHP behaviors in relation to delivery of CCC regardless of setting (*e.g.*, VA, DOD, or academic and community health centers). By adopting a systematic approach to achieving expert consensus, our aim was to ensure the likelihood of developing a measure high in content validity that could inform CCC implementation efforts and be acceptable to both administrators and frontline BHPs.

## Methods

### Fidelity framework

Fidelity is the most commonly reported measure of implementation outcomes [29]. The US National Institutes of Health Behavior Change Consortium has described five aspects of fidelity as follows: study design, provider training, treatment delivery, treatment receipt, and treatment enactment [30]. Although all of these aspects of fidelity can ultimately influence implementation and outcomes of a program, evaluation of treatment delivery is often prioritized to verify if an intervention is being provided as intended [31]. As described by Perepletchikova and Kazdin [32], fidelity in treatment delivery can be further specified as provider adherence (*i.e.*, utilization of specified procedures and engagement in specific tasks and activities) and treatment differentiation (*i.e.*, care that is reflective of critical dimensions of CCC rather than specialty mental healthcare). We chose to emphasize adherence and differentiation over other aspects of delivery, such as provider competence (*i.e.*, skillfulness in delivering treatment), given the multitude of factors that might impact the skill set of BHPs and to avoid blurring fidelity assessment and individual performance appraisal.

Having identified adherence as the most relevant focus for our investigation of BHP fidelity, our conceptual framework for this study was guided by Carroll *et al.*[33], who described intervention adherence as the “bottom line” of implementation fidelity. Carroll *et al.*[33] suggest that adherence mediates the impact of an intervention on outcomes, with adherence reflecting how much of an intervention’s content, or active ingredient, is delivered. Estimates of adherence are quantifiable, based on the frequency, duration, or coverage of the intervention content. Furthermore, some intervention content may be prioritized over other content if it is considered ‘essential,’ or required for the intervention to produce the expected outcome. This conceptualization of fidelity guided the specific study approach described below.

### Modified Delphi study approach

The modified Delphi approach refers to a structured, iterative process of collecting and summarizing opinions from content experts with the primary goal of consensus building [34-36]. This approach is often used to address problems of clinical practice when there is incomplete knowledge on a subject [37,38]. The modified Delphi process polls a panel of experts who provide feedback about an evolving set of statements during several rounds of data collection. As applied to the current instrument development study, the Delphi method helps ensure content validity and an appropriate item pool that sufficiently represents behaviors of BHPs in CCC. We employed three rounds of data collection with the aim of reaching at least 80% consensus among participants on each item [34]. Participants were asked to re-rate items that had less than 80% consensus after round one, providing a process by which participants could anonymously agree or disagree with other experts. An advantage to this approach is that it provides anonymity, thereby decreasing the likelihood that some participants will modify their responses based on the opinions of highly influential experts [35].

### Participants

Purposeful sampling was used to recruit VA and non-VA experts in CCC known to the study investigators as expert clinicians, administrative leads, or content experts based on previously published research. Although panel sizes for Delphi studies vary considerably depending on the goal of the project and availability of experts [36], we invited 33 experts to participate with the goal of recruiting at least 20 experts through three rounds of data collection. In round one, 88% (n = 29) of experts contacted returned completed surveys. In round two, 86% (n = 25) of round one participants returned completed surveys. In round three, 100% (n = 25) of round two participants returned completed surveys yielding a 91% response rate across rounds. Thus, the overall response rate for those participating in the entire study was 76%.

### Survey item development

Guided by the fidelity framework [33] and related literature [19,32] noted above, initial survey items were generated and refined over a six-month period by the study team. Items were developed to represent the critical components of the CCC model enacted by BHPs based on a qualitative review of published scientific literature [1,6-8,12,13,24-26,39-41], unpublished clinical practice guidelines from clinics using a CCC platform, clinical and administrative experience of the research team, and informal polling of CCC experts known to the research team but not asked to be part of the Delphi study [19,42]. The CCC-related publications reviewed were in-depth descriptions of CCC model aims, procedures, and components because empirical studies relating CCC components to clinical outcomes have not been conducted. We therefore translated these descriptions of CCC models into a pool of items representing discrete BHP behaviors so that expert opinion could be used to evaluate the relevance of each behavior within the conceptual domain of BHP protocol adherence. To ensure breadth in the development of the items, several aspects were considered. First, we attempted to identify components that reflected either BHP behavior related to the delivery of clinical services to patients or related to collaboration with the primary care team. Second, items were classified as representing either structural elements of care (the framework for CCC service delivery) or process elements of care (the methods or procedures related to CCC service delivery [19]). Third, subgroups of the final set of items were classified by content domains representing core components of BHP practice as determined by the research team: clinical scope and interventions; practice and session management; referral management and care continuity; consultation, collaboration, and interprofessional communication. Fourth, items were further classified by the study team as essential, compatible, or prohibited when working in CCC [31]. Essential behaviors were those considered to be highly reflective of the CCC model and required for good practice, even if that behavior did not necessarily need to happen in every encounter. Compatible behaviors were those that were acceptable when working in a CCC model, but are not required or specific to CCC. Prohibited items were those behaviors that should be clearly avoided when working in a CCC model because they were inconsistent with CCC.

### Survey materials

The round one survey included a brief introduction to the purposes and goals of the study followed by a definition of CCC to help orient participants to the general model of behavioral health in primary care. As noted previously, nomenclature and definitions of CCC-type models of care vary. Thus, the definition of CCC provided to participants was selected from the VA Center for Integrated Healthcare Clinical Operations Manual [43]:

‘Co-located, Collaborative Care services are offered by an embedded behavioral health provider. This approach involves providing services to primary care patients in a collaborative framework within primary care teams. Behavioral health visits are brief (generally 20–30 minutes), limited in number (1–6 visits with an average of between 2 and 3), and are provided in the primary care practice area, structured so that the patient views meeting with the behavioral health provider as a routine primary care service and medical providers are supported across a broad scope of behavioral health concerns.’

Note that we did not provide any additional review of published CCC literature because the study investigators felt this information was sufficiently captured in the content of the item pool. Participants were asked to classify the 56 items in the round one survey that reflected BHP behaviors related to the CCC model as essential, compatible, or prohibited when working in CCC. Open-ended questions followed each item so that participants could suggest modifications to the item and provide a rationale for suggested changes. Additionally, several open-ended items at the end of the survey requested information on what BHP behaviors should be added to the item pool. Finally, participants were asked to report professional background characteristics, such as their primary occupational setting.

The round two survey was similar to the round one survey, but was modified to reflect round one findings. First, participants were asked to comment on and indicate if they agreed (yes/no) with a revised definition of CCC. Second, participants were presented with 37 items from round one that did not meet sufficient consensus. To aid participants in re-rating these items, they were reminded of their original response to the item and shown the current level of consensus (*i.e.*, the aggregated responses from round one). Additionally, two new items that were suggested in the open-ended responses of round one were added for a total of 39 items. Third, participants were also provided, for informational purposes only, the 19 items that met consensus in round one.

**Figure 1:**
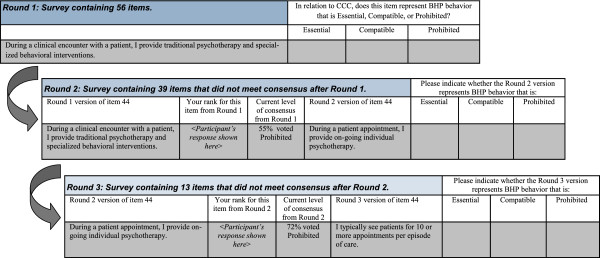
Example of presentation of survey items across three rounds of the Delphi study.

The round three survey was modified to reflect the results from round two. First, participants were asked to comment on and indicate their agreement (yes/no) with a subsequently revised definition of CCC. We provided an additional opportunity to modify the CCC definition and label because qualitative feedback in round two pointed to concerns that ‘co-located, collaborative care’ was an unclear term. Therefore, we asked respondents to select their preferred label for the model described. Second, participants were asked to rate for a final time the remaining 13 items that did not reach consensus after round two. Third, participants were presented the 26 items that met consensus after round two for informational purposes only. An example of how items were presented to participants across rounds is provided in Figure 1.

### Procedures

All communication between the research team and participants was conducted by email. The study PI (GPB) and Co-I (JSF), both clinical research psychologists at the VA Center for Integrated Healthcare were primary contacts for the study and initiated all email correspondence. All emails were sent to individuals, rather than the full group of participants, from the investigators’ personal VA email accounts to maintain privacy. Potential participants first received an email briefly introducing the purpose of the study and nature of their participation, including the goal of building consensus with other experts by completing up to three surveys. Attached to this email was a document that included informed consent information and the round one survey. Note that we sought and were granted a waiver of written informed consent from our Institutional Review Board because of the low-risk nature of the study and the goal of increasing feasibility by reducing response burden to the greatest extent possible. Experts were asked to read the consent document, contact the study team with any questions or concerns as necessary, and continue on to complete the survey to indicate their agreement to participate. To encourage participation, up to three personalized email reminders were sent per round. When participants returned their completed survey by email, they were provided with a thank you reply along with information indicating that they would be contacted within several weeks with the round two survey comprised of an updated survey reflective of round one findings. Similar information was provided to participants when they completed round two surveys in anticipation of round three. Those participants who ultimately did not complete the round one survey were not contacted to complete the round two survey. All participants who completed round two subsequently completed round three. The conduct of this study was approved by the VA Western New York Healthcare System Research & Development Committee and Institutional Review Board.

### Data analysis

Descriptive statistics (frequencies, percentages) were calculated for each item after each round of surveys were completed. Brief qualitative responses to open-ended questions were summarized by item and reviewed by the research team to search for consistencies to aid the interpretation of the quantitative findings. The research team each reviewed the qualitative data separately and then discussed summaries of findings together to reach consensus regarding main concerns or queries raised by participants following each round. Those items with 80% or greater agreement in one category (*i.e.*, essential, compatible, or prohibited) were considered to have met consensus and were only modified to address minor word choice or grammatical errors as needed. Those items with less than 80% consensus were either modified to incorporate participants’ feedback provided in qualitative responses or presented again in a subsequent round without modification. Items that failed to reach consensus after round three were eliminated from the item pool.

## Results

### Participant characteristics

As shown in Table 1, self-reported professional background among participants that completed all three rounds (n = 25) confirmed that the sample met the goal of reaching clinical, administrative, and research experts with extensive knowledge of CCC. Participants were primarily doctoral-level psychologists (92%), current or former BHPs in CCC (96%), and held an administrative leadership position related to the implementation of CCC in their institution (68%). Experts’ primary occupational setting was VA (40%), DOD (28%), or other (32%), including academic or community health centers. Many participants had authored or co-authored a peer-reviewed publication that was an empirical investigation related to CCC (46%) or a descriptive paper regarding CCC models (55%). In examining the characteristics of the non-responders, seven of eight non-responders were psychologists and the remaining non-responder was a physician. Two non-responders had primary affiliations with the DOD, three non-responders were affiliated with VA, and three non-responders were affiliated with academic health centers suggesting adequate representation across settings.

**Table 1:** **Delphi study participants completing three rounds of surveys (*n *= 25)**

**Variable**		***n*(%)**
Educational background		
	MD/DO	1 (4)
	PhD/PsyD	23 (92)
	NP	1 (4)
Primary occupational setting		
	VA	10 (40)
	DOD	7 (28)
	Other	8 (32)
Behavioral Health Provider in CCC		
	Yes	24 (96)
	No	1 (4)
Years	Median: 9	
	Range: 2-27	
Primary Medical Provider in CCC		
	Yes	0
	No	25 (100)
Administrative lead for implementing CCC		
	Yes	17 (68)
	No	8 (32)
Author/Co-author of peer reviewed paper regarding CCC*		
Empirical research	Yes	11 (46)
	No	13 (54)
Other paper	Yes	13 (54)
	No	11 (46)

*n=24 due to missing data.

Note: CCC = co-located, collaborative care.

### Model definition and preferred terminology

Based on participant feedback, the original definition of CCC presented in survey round one was expanded considerably, with 84% of participants agreeing on the final definition presented below. Modifications to the original definition are bolded:

‘Co-located, Collaborative Care (CCC) services are offered by an embedded Behavioral Health Provider. This approach involves providing **patient-centered** behavioral health services to primary care patients in a collaborative framework within primary care teams **that utilize a shared medical record**. Behavioral health appointments are usually brief (**30 minutes or less**), **typically limited in number** (**1–6 visits with an average of between 2 and 3 per care episode**), and are provided in the primary care practice area. These appointments are structured so that the patient views meeting with the Behavioral Health Provider as a routine primary care service. **Primary Care Providers maintain responsibility for patient care decisions**, but are supported by the Behavioral Health Providers who **emphasize patient self management** across a broad scope of concerns, **including common mental health diagnoses, physical health issues, and prevention. CCC Behavioral Health Providers may also coordinate, or blend, their services with other forms of primary care based behavioral health (*e.g.,* depression Care Management).’**

Although modifications to the definition led to agreement about the general features of the model, the term ‘co-located, collaborative care’ was not well-agreed upon. Participants ultimately preferred the term ‘primary care behavioral health’ (41.7%) over ‘co-located, collaborative care’ (16.7%), ‘integrated primary care’ (29.2%), or other terms (‘collaborative care’ or ‘integrated behavioral health’ 12.5%).

### Delphi process findings

Table 2 displays the original item wording, component domains, classification applied by the study team, final item wording, and the consensus rating following round three. Following round one of the Delphi process, 19 survey items met consensus, leaving 37 items to be rated again in round two. All but one item (item 35) was modified for the round two survey based on qualitative feedback received. Most changes to items were focused on improving clarity, eliminating terms or acronyms that were used exclusively within VA settings, or adding qualifiers (such as ‘typically’) in describing certain behaviors. Other changes were more substantive, such as modifying item 18’s emphasis on the content of progress note documentation to the time spent on documentation. Additionally, two new items suggested by participants in round one were constructed by the study team to address providing feedback to primary care teams about initiating or modifying psychotropic medications (item 57) and participation in clinical pathways for common health conditions (item 58) for a total of 39 items to be rated in round two.

**Table 2:** Item content, content domains, and consensus ratings of the Primary Care Behavioral Health Provider Adherence Questionnaire (PPAQ)

**Original Item**	**Content domain**	**Research team rating**	**Final item, if modified from original**	**Delphi rating**
1. During clinical encounters with patients, I see patients for 30 minutes or less.	Practice and Session Management	Essential	N/A	83% Essential
2. I manage patients reporting mild and moderate symptoms and refer those with more severe symptoms to other behavioral health services.	Practice and Session Management	Essential	2. I manage patients reporting mild and moderate symptoms in primary care, and I refer those with more severe symptoms to specialty mental health services when possible.	88% Essential
3. During clinical encounters with a patient, I discuss barriers to implementing a plan or adhering to treatment recommendations.	Practice and Session Management	Essential	3. During patient appointments, I discuss barriers to implementing a plan or adhering to treatment recommendations.	80% Essential
4. I collaborate with primary care team or PACT staff to provide group medical visits to patients	Consultation, Collaboration, and interprofessional communication	Compatible	4. I collaborate with primary care team staff to provide group medical visits (or shared medical appointments) to patients.	80% Compatible
5. I accept referrals for patients with traditional mental health problems (*i.e.* depression, anxiety, PTSD, etc.).	Referral management and care continuity	Essential	5. I accept referrals for patients with common mental health problems (*i.e.* depression, anxiety, etc.).	80% Essential
6. During clinical encounters with a patient, I implement behavioral and/or cognitive interventions.	Clinical scope and Interventions	Essential	N/A	86% Essential
7. In introducing my role in the clinic to patients, I explain that I want to get an idea of what is and what is not working for the patient and then together develop a plan to help them manage their concerns.	Practice and Session Management	Essential	7. In introducing my role in the clinic to patients, I explain that I want to get an idea of what is and what is not working for the patient and then together develop a plan to help them manage their concerns.	80% Essential
8. During clinical encounters with patients, I triage patients to determine if they can be treated in primary care or should be referred to a specialty mental health or a community agency.	Practice and Session Management	Essential	N/A	86% Essential
9. I accept referrals for patients in need of behavioral health interventions for chronic pain.	Referral Management and Care Continuity	Essential	9. I accept referrals for patients who might benefit from brief, targeted behavioral health interventions for chronic pain.	80% Essential
10. I accept referrals for patients in need of behavioral health interventions for adjustment to illness (*i.e.*, diabetes, heart disease, spinal cord injury, TBI, etc.).	Referral Management and Care Continuity	Essential	10. I accept referrals for patients who might benefit from brief, targeted behavioral health interventions for adjustment to illness (*i.e.*, diabetes, heart disease, spinal cord injury, TBI, etc.).	96% Essential
11. My progress notes include focused recommendations for the PCP	Practice and Session Management	Essential	11. My progress notes in the shared medical record include focused recommendations for the Primary Care Provider and/or primary care team.	88% Essential
12. I huddle with the primary care team or PACT staff to provide both a behavioral health perspective and behavioral data.	Consultation, Collaboration, and interprofessional communication	Essential	12. I meet briefly with primary care staff as a team to provide both a behavioral health perspective and behavioral data.	88% Essential
13. My progress notes include focused recommendations for the patient.	Practice and Session Management	Essential	N/A	86% Essential
14. During clinical encounters with patients, I provide educational handouts to the majority of patients.	Clinical Scope and Interventions	Essential	14. During patient appointments, I provide educational handouts when appropriate.	72% Compatible
15. I routinely consult with primary care team or PACT staff other than the PCP (*i.e.*, pharmacist, dietician) about behavioral aspects of medical conditions (*i.e.*, medications that cause nightmares.)	Consultation, Collaboration, and interprofessional communication	Essential	15. I consult with various members of the primary care team (*i.e.*, pharmacist, dietician) in addition to the Primary Care Provider about behavioral aspects of medical conditions (*i.e.*, medications that cause nightmares.)	80% Essential
16. At follow-up encounters with patients, I inquire about progress on goals or action plans set at the previous appointment.	Practice and Session Management	Essential	N/A	90% Essential
17. During clinical encounters with patients, I routinely complete standardized measures for an initial screening (*e.g.*, PHQ-9, PCL, or brief cognitive screening).	Practice and Session Management	Essential	17. I administer one or more brief validated measures (*e.g.*, Patient Health Questionnaire-9, or PHQ-9) for an initial screening of symptoms of interest, or I review these findings if measures were administered by other primary care staff.	84% Essential
18. I document a full-length treatment plan with multi-axial diagnosis after the initial encounter.	Practice and Session Management	Prohibited	18. It takes 30 minutes or more for me to complete all documentation following the initial appointment.	80% Prohibited
19. During a clinical encounter with a patient, I use reflection of affect and silence to promote emotional exploration.	Clinical Scope and Interventions	Prohibited	19. During patient appointments, I promote emotional exploration.	88% Compatible
20. Following clinical encounters with patients, I provide feedback to the PCP within 1 business day of initial patient contact.	Consultation, Collaboration, and interprofessional communication	Essential	20. Following patient appointments, I provide feedback to Primary Care Providers (based on their preferred method of communication) within 1 business day of an initial appointment.	96% Essential
21. During clinical encounters with patients, I clarify, confirm, and discuss the patient’s concerns.	Practice and Session Management	Essential	N/A	86% Essential
22. My progress notes include a brief clinical conceptualization or impressions.	Practice and Session Management	Essential	22. My progress notes include brief clinical impressions of the patient’s presenting problem(s).	80% Essential
23. During a clinical encounter with a patient, I provide full neuropsychological, cognitive, or personality assessments.	Clinical Scope and Interventions	Prohibited	N/A	96% Prohibited
24. I see patients for weekly, open-ended therapy.	Practice and Session Management	Prohibited	N/A	86% Prohibited
25. In introducing my role in the clinic to patients, I explain that our sessions will be less than 30 minutes.	Practice and Session Management	Essential	25. In introducing my role in the clinic to patients, I explain that our appointments typically will be 30 minutes or less.	84% Essential
26. I provide behavioral health crisis or emergency intervention (*i.e.* suicide intervention) as the CCC BHP.	Clinical Scope and Interventions	Compatible	26. I provide suicide risk assessment for primary care patients in crisis and refer to a higher level of care as indicated.	84% Essential
27. During clinical encounters with patients, I see patients for 50-minute appointment.	Practice and Session Management	Prohibited	27. I typically see patients for 50-minute appointments.	80% Prohibited
28. During clinical encounters with patients, I use local community resources to assist me in meeting the behavioral health needs of patients.	Practice and Session Management	Essential	28 During patient appointments, I use local community resources to assist me in meeting the behavioral health needs of patients.	84% Essential
29. I provide education to the primary care team or PACT staff on behavioral health issues (*e.g.*, presentations and handouts).	Consultation, Collaboration, and interprofessional communication	Essential	N/A	86% Essential
30. I provide advice to primary care team or PACT staff about appropriate referrals to specialty behavioral health services.	Referral Management and Care Continuity	Essential	N/A	86% Essential
31. I conduct follow-up sessions via telephone.	Practice and Session Management	Compatible	31. I conduct follow-up appointments via telephone when appropriate.	88% Compatible
32. During a clinical encounter with a patient, I provide traditional family therapy to patients and couples.	Clinical Scope and Interventions	Prohibited	32. I provide family or couples therapy for 10 or more appointments per episode of care.	84% Prohibited
33. During a clinical encounter with a patient, I primarily use open-ended questions.	Clinical Scope and Interventions	Prohibited	33. During patient appointments, I use open-ended questions.	72% Compatible
34. On average, I see patients for only 2–3 consultations.	Practice and Session Management	Essential	34. I typically see patients for 6 or less appointments per episode of care.	79% Essential
35. My progress notes include findings from functional assessments and brief screening instruments.	Practice and Session Management	Essential	N/A	96% Essential
36. During clinical encounters with patients, I complete standardized measures for assessing change at follow up (*e.g.*, PHQ-9, PCL, or brief cognitive screening).	Practice and Session Management	Essential	36. I administer one or more brief validated measures (*e.g.*, Patient Health Questionnaire-9, or PHQ-9) for follow up screening of symptoms of interest, or I review these findings if measures were administered by other primary care staff.	84% Essential
37. I routinely consult with PCPs to increase my knowledge about behavioral aspects of medical conditions, such as the role of anxiety in cardiac distress.	Consultation, Collaboration, and interprofessional communication	Essential	N/A	83% Essential
38. During a clinical encounter with a patient, I provide supportive interventions without addressing cognitive or behavioral change.	Clinical Scope and Interventions	Compatible	38. During a patient appointment, I provide supportive interventions without addressing cognitive or behavioral change.	84% Compatible
39. During a clinical encounter with a patient, I provide full-length empirically supported treatments (ESTs), such as Prolonged Exposure or Cognitive Processing Therapy.	Clinical Scope and Interventions	Prohibited	39. During a patient appointment, I provide full-length empirically supported treatments, such as Prolonged Exposure or Dialectical Behavior Therapy.	88% Prohibited
40. Following clinical encounters with patients, I continue to provide feedback to the PCP about follow-up appointments when needed.	Consultation, Collaboration, and interprofessional communication	Essential	N/A	90% Essential
41. During clinical encounters with patients, I work with the patient to develop a specific plan to address their presenting problem and document this plan.	Practice and Session Management	Essential	N/A	83% Essential
42. I accept referrals for patients who need lifestyle interventions (*e.g.*, tobacco cessation, weight control, stress management).	Referral Management and Care Continuity	Essential	N/A	97% Essential
43. I accept referrals for patients in need of behavioral health interventions for medication issues (*i.e.*, adherence).	Referral Management and Care Continuity	Essential	N/A	86% Essential
44. During a clinical encounter with a patient, I provide traditional psychotherapy and specialized behavioral interventions.	Clinical Scope and Interventions	Prohibited	44. I typically see patients for 10 or more appointments per episode of care.	88% Prohibited
45. Following clinical encounters with patients, I schedule follow-ups at least two weeks apart.	Practice and session management	Essential	45. Following patient appointments, I typically schedule follow-ups at least two weeks apart.	84% Compatible
46. I accept referrals for patients in need of behavioral health interventions for adjustment to aging and issues specific to older patients.	Referral Management and Care Continuity	Essential	46. I accept referrals for patients in need of behavioral health interventions for adjustment to aging and issues specific to older patients.	80% Essential
47. During a clinical encounter with a patient, I provide a highly structured encounter to address functional assessment, focused intervention, and disposition.	Practice and Session Management	Essential	47. During a patient appointment, I provide functional assessment, focused intervention, and address disposition.	92% Essential
48. I provide brief psycho-education and symptom management groups as part of my role as CCC BHP.	Clinical Scope and Interventions	Compatible	48. I provide brief psycho-educational groups or classes on specific topics (such as mood management, stress reduction, etc.).	80% Compatible
49. During a clinical encounter with a patient, I provide brief consultation to couples or families.	Clinical Scope and Interventions	Compatible	49. I have appointments with couples and families as appropriate.	68% Compatible
50. I accept referrals for patients from PCPs as a warm hand off (*i.e.*, the PCP introduces me to the Veteran).	Referral management and Care continuity	Essential	N/A	100% Essential
51. In introducing my role in the clinic to patients, I explain that I work with the PCPs in situations where good healthcare involves paying attention to physical health, habits, behaviors, emotional health and how those things interact.	Practice and Session Management	Essential	N/A	83% Essential
52. I provide long-term (*i.e.*, greater than 6 sessions) group psychotherapy, such as DBT, as part of my role as CCC BHP.	Clinical Scope and Interventions	Prohibited	52. I provide long-term (*i.e.*, greater than 8 sessions) group psychotherapy.	80% Prohibited
53. During a clinical encounter with a patient, I obtain a full psycho-social history.	Clinical scope and Interventions	Prohibited	53. I meet with a patient for greater than 50 minutes to gather a full psycho-social history and comprehensive psychiatric interview.	88% Prohibited
54. During a clinical encounter with a patient, I provide medical social work services.	Clinical Scope and Interventions	Prohibited	54. During a patient appointment, I typically provide medical social work services, including, but not limited to, assistance with disability claims, obtaining health insurance, and/or assisting with housing.	88% Prohibited
55. During clinical encounters with patients, I address the PCPs reason for referral.	Practice and Session Management	Essential	N/A	93% Essential
56. I employ strategies to identify and prevent exacerbation of at-risk, sub-syndromal behaviors and symptoms.	Clinical Scope and Interventions	Essential	N/A	83% Essential
57. *Not included in Round 1 Survey*	Consultation, Collaboration, and interprofessional communication		57. I provide information regarding a patient’s symptoms and functioning to assist Primary Care Providers (and/or clinical pharmacists, primary care psychiatrists, psychiatric nurse practitioners) in initiating or modifying common psychotropic medications, such as antidepressants.	84% Essential
58. *Not included in Round 1 Survey*	Clinical Scope and Interventions		58. I participate in primary care based clinical pathways for common health conditions, such as chronic pain or comorbid depression and cardiovascular disease. *A clinical pathway is an approach to managing patients with common conditions by utilizing empirically supported interventions in a pre-defined sequence among a multidisciplinary group of providers.*	80% Essential

**Notes:** Items 57 and 58 were created during the Delphi process based on qualitative feedback suggesting new items were necessary. Content domain labels for these items were subsequently applied by the research team.

At the end of round two, 26 of the 39 items met consensus, leaving only 13 items for round three. Eight of the 13 items in round three were again modified based on qualitative feedback, but no additional items were added to the survey. At the end of round three, 54 items (93%) from the 58 total items across rounds met sufficient consensus to be included in the final survey. The four items that failed to reach consensus (items 14, 33, 34, 49) had final rankings that ranged from 68.0% to 79.2% agreement and covered a range of behaviors. Our ability to explain the lack of consensus for these items was limited due to our small sample size. However, when we inspected response patterns by primary occupational setting, VA respondents tended to consider item 14 (use of educational handouts) to be more essential (40.0%) than respondents from DOD (28.6%) or other settings (12.5%). For item 33 (use of open-ended questions), VA respondents tended to classify this behavior as prohibited (30.0%) more frequently than participants from DOD (0.0%) or other settings (12.5%). For item 34 (seeing patients for 6 sessions or less), VA participants (90.0%) and DOD participants (85.7%) tended to rate this item as essential more frequently than participants from other settings (50.0%). Finally, for item 49 (having appointments with couples/families), participants from other settings were more likely to view this behavior as essential (62.5%) compared those participants from VA (20.0%) or DOD (14.3%).

### Final item ratings

Overall, 54 items comprised the final survey which was named the Primary Care Behavioral Health Provider Adherence Questionnaire, or PPAQ*,* by the study team, reflecting the participants’ preference for the term ‘primary care behavioral health’ over ‘co-located, collaborative care.’ There were 24 items representing practice and session management, 14 items representing clinical scope and interventions, eight items representing referral management and care continuity, and eight items representing consultation, collaboration, and interprofessional communication. Based on final ratings, there were 38 essential items, 10 prohibited items, and six compatible items in the PPAQ. Final ratings typically matched the initial ratings applied by the study team, with the exception of items 19, 26, and 45. These discrepancies appear largely due to substantive changes made to the items based on qualitative feedback during the Delphi process. Note that items 57 and 58 did not receive initial ratings by the study team because they were added during the Delphi process based on participant feedback.

## Discussion

A vital element of ensuring high quality and empirically based intervention for integrated healthcare is the availability of valid fidelity measures to provide a common metric of implementation. The present study followed a systematic approach to develop a measure of fidelity, the PPAQ, to a popular model of integrated healthcare, CCC. Filling a significant gap in the literature, the PPAQ provides an adherence assessment tool focused on the BHP’s behavior within CCC which represents a significant first step in developing measurement for comprehensive assessment of CCC fidelity of implementation.

The present study was successful in obtaining a high response rate across the three rounds of data collection from the panel of CCC experts. Experts were from diverse settings, which ensured a representative range of responses. In addition, a majority of the experts practiced within this type of setting for several years demonstrating hands-on knowledge of the BHP’s roles within CCC. Ninety-three percent of the original items met the 80% consensus cut-off rate yielding the final 54 items that represented the four content domains originally conceptualized by the research team. A majority of the items included on the PPAQ belong to the practice and session management, clinical scope, and interventions domains. This finding is not surprising because previously published CCC literature [6,43] focuses heavily on the population-based framework of the model that emphasizes providing easily accessible care to a large number of patients with a wide range of acute, chronic, and preventive medicine concerns [14]. Providing population-based care impacts BHP clinical behaviors directed toward a brief, time-limited treatment model compared to specialty mental health settings which do not follow that framework. For example, a key behavior related to session management is represented in item 1, which was rated as an essential component and emphasizes that session length should be 30 minutes or less.

The final PPAQ included a mixture of essential, compatible, and prohibited items [32]. Including prohibited items ensured that this instrument could provide information on whether or not BHP’s behavior is in stark contrast to what would be expected based on the theoretical foundations of CCC. Prohibited items focus on elements of practice and session management as well as clinical scope and interventions that would likely be more consistent with BHPs working within specialty mental health setting rather than primary care. Notably, the low number of prohibited items also reflects the CCC experts’ acknowledgement that this model needs to be flexible, patient-centered, and adaptable to the clinic setting. Therefore, using the PPAQ to monitor prohibited items that reflect highly time-consuming activities (such as conducting weekly, open-ended therapy) provides an opportunity to correct some of the most critical BHP behaviors that could limit their ability to enact a population-based model. Similarly, the compatible items also reflected the CCC experts’ inherent acknowledgement of the need for flexibility within the CCC model. Identification of compatible items, such as participating in group medical visits, are important in that they specify activities that are not required for CCC practice, but are acceptable within the model.

The four items that did not reach consensus appeared to be significantly affected by the type of setting in which the CCC expert worked, suggesting that these items are potentially context-dependent or related to implementing CCC in settings with unique clinic characteristics or particular programmatic goals. Although employing a larger expert panel may have made it easier to reach consensus on these items, the value of the PPAQ is that the items were developed to reflect universal BHP behaviors. Future research will need to examine the system, clinic, and patient factors specifically related to CCC that may influence both provider adherence and the nature of the intervention. These moderators, such as participants’ responsiveness, represent a variety of real world contextual factors and are important to consider when conducting implementation research [33].

The expert panel also provided a revised definition of the CCC model. This definition is vital, because the field of integrated healthcare has been confounded by different definitions and terms [1]. The definition of CCC agreed upon by the expert panel is consistent with the nature of the items included in the PPAQ, with a fundamental focus on providing population-level care. For example, the expert panel’s consensus definition included specific limits to the length and number of sessions due to its importance in the CCC model. Unlike previously published definitions, this definition of CCC is the only one that we are aware of that has been empirically derived through a systematic process of gathering opinions from a diverse group of experts to produced a definition that might be less biased than those from investigator teams representing single institutions or systems. Although the majority of the panel preferred the name ‘primary care behavioral health’ for the model, the panel as a whole was unable to reach a high level of consensus. This lack of consensus may reflect participants’ preference for using terms unique to their healthcare system. Therefore, although this study was able to provide a useful model definition, it was unable to fully resolve the ongoing difficulties within the field regarding specific terminology.

Although additional study of the PPAQ is necessary, CCC stakeholders may find it useful in its current form for guiding BHPs who are new to the CCC setting. Formal graduate or post-doctoral training in CCC models is scarce [41,44], yet transitioning to the CCC setting requires skill in differentiating between behaviors that are reflective of population-based care compared to traditional specialty mental healthcare. We are not aware of any instruments to date that specify particular behaviors that can be used to help BHPs discriminate among behaviors essential for maintaining key CCC programmatic goals. The PPAQ can therefore be used by clinic administrators, clinical supervisors, or program facilitators as part of an educational process about the roles and functions of BHPs. Attention to total and domain-specific PPAQ scores, as well as analysis of individual items, would also suggest areas where provider-focused implementation support is required. Although the PPAQ’s sensitivity to change over time has yet to be investigated, because it includes items that are fundamentally important to the function of CCC, it may also prove helpful beyond initial training and implementation stages to assess program maintenance.

### Strengths and limitations

The present study is the first step towards establishing the PPAQ as a useful fidelity assessment tool of the CCC model. The high participation rate among a panel of experts and high level of consensus required (≥80%) help support the PPAQ content validity. However, this study does have some limitations. Foremost, results of this study are based primarily on expert opinion because there is little empirical evidence regarding which components of CCC, operationalized as BHP behaviors, are predictive of clinical outcomes. Thus, this study represents a first step in providing a tool for future dismantling studies that can evaluate empirically which aspects of protocol adherence (*i.e.*, combinations of items within or across essential, compatible, or prohibited categories) are related to patient outcomes. The PPAQ was also developed to focus only on the adherence of BHPs to the CCC model and does not assess fidelity at a system- or patient-level [29]. We made this choice for several reasons. Contextual factors in the conceptual framework of fidelity we adopted [33] are proposed to impact outcomes only indirectly, as moderators, through provider adherence. Therefore, it seemed logical to first quantify provider intervention adherence because of its direct and proximal association with clinical outcomes. Due to our interests in expanding the evidence base for integrated healthcare, we also prioritized the development of a measure of adherence that could address the increasing pressure for clinical outcome data demonstrating the effectiveness of specific formulations of CCC. Additionally, as noted previously, we aimed to develop an efficient and easy-to-use measure to guide implementation of CCC using BHP self-report.

Whereas we included BHPs in the development of the PPAQ, more formal study of provider reactions to and acceptability of the measure is warranted. Future studies will need to consider the degree to which social desirability plays a role in shaping their responses. For example, BHPs may feel they need to report high levels of fidelity by either over-reporting essential behaviors and/or under-reporting prohibited behaviors if they believe that their report is linked to individual performance evaluation. Therefore, if the PPAQ was being used for training and program development, it would be important to provide education to BHPs regarding the purpose of fidelity assessment as a learning opportunity and to remain supportive when introducing the PPAQ to establish a norm of routine fidelity assessment. When used in future wider-scale research on patient outcomes, rather than as a tool for training, the nature of the PPAQ as a self-administered instrument may be beneficial for reducing the impact of social desirability [45], particularly if the research design allows for administration over the internet or other approaches that preserve respondent anonymity.

Although this study demonstrates that the PPAQ items are content valid, future research needs to be conducted to establish the reliability and other aspects of validity. For example, although the PPAQ was designed to measure BHP behaviors in CCC, we did not examine whether the PPAQ is able to discriminate CCC from other models of integrated healthcare, such as care management [46,47] or to predict CCC outcomes. While necessary, this type of work may be difficult to conduct due to the small number of BHPs working in these settings, the difficulty of identifying and accessing providers, and the large sample size necessary to conduct the traditional factor analysis that typically is used when validating assessment measures. However, an investigation to examine the reliability and validity of the PPAQ with BHPs is currently underway by the research team.

## Conclusions

Developing a psychometrically sound fidelity tool is imperative as integrated healthcare continues to become more pervasive in the US and internationally. The creation of the PPAQ is one of the first steps towards initiating this process as researchers, evaluators, and administrators search for ways to assess the implementation of CCC. Greater attention to the role of BHP behavior in CCC serves two related purposes: a viable approach to monitoring and assessing how CCC is implemented in real-world clinics, and identifying areas where the development of provider-centric facilitation support is necessary. Offering better support to BHPs as they provide behavioral health services at the frontline of the healthcare system may ultimately pave the way for improved implementation and long-term sustainability of integrated care.

## Abbreviations

BHP: Behavioral Health Provider; CCC: Co-located, Collaborative Care; DOD: Department of Defense; PPAQ: Primary Care-Behavioral Health Provider Adherence Questionnaire; PC-MHI: Primary Care-Mental Health Integration; PCP: Primary Care Provider; US: United States; VA: Veterans Administration

## Competing interests

There are no known conflicts of interest for reasons financial or otherwise, no known competing interests, and no companies or products are being featured in this research.

## Authors’ contributions

GB conceived of the study, and GB, JF, and KP developed its design. All authors contributed to the acquisition of data. GB and CV conducted the primary quantitative data analysis. All authors contributed to the qualitative analysis and contributed to the interpretation of data. GB, JF, and KP drafted the manuscript. CV assisted in critically revising the manuscript. All authors read and approved the final manuscript.

## Authors’ information

Requests for the Primary Care Behavioral Health Provider Adherence Questionnaire (PPAQ), including scoring information, can be directed to the corresponding author.
